# Supplementary material for: Mechanisms governing the pioneering and redistribution capabilities of the non-classical pioneer PU.1
Source: Nat Commun. 2020 Jan 21;11:402. doi: 10.1038/s41467-019-13960-2 (PMC6972792; doi:10.1038/s41467-019-13960-2)
Supplement: Supplementary file 7 — Source data [file 41467_2019_13960_MOESM7_ESM.zip › Source_Data/FigureS2/FigureS2B_MotifScanOutput/KmeansCluster4/homerResults/motif27.similar.html]

motif27

## Information for motif27

A
T
C
G
A
C
G
T
T
C
A
G
C
G
T
A
A
C
T
G
A
C
T
G
A
G
T
C
G
T
A
C
A
C
G
T
G
A
T
C
  
Reverse Opposite:  

C
T
A
G
T
G
C
A
C
A
T
G
T
C
A
G
T
G
A
C
T
G
A
C
G
C
A
T
A
G
T
C
T
G
C
A
T
A
G
C
  

|  |  |
| --- | --- |
| p-value: | 1e-37 |
| log p-value: | -8.624e+01 |
| Information Content per bp: | 1.511 |
| Number of Target Sequences with motif | 171.0 |
| Percentage of Target Sequences with motif | 3.79% |
| Number of Background Sequences with motif | 437.5 |
| Percentage of Background Sequences with motif | 1.05% |
| Average Position of motif in Targets | 90.2 +/- 70.5bp |
| Average Position of motif in Background | 101.7 +/- 71.0bp |
| Strand Bias (log2 ratio + to - strand density) | 0.0 |
| Multiplicity (# of sites on avg that occur together) | 1.05 |
| Motif File: | file (matrix) reverse opposite |

### Similar de novo motifs found

|  |  |  |  |  |  |  |  |
| --- | --- | --- | --- | --- | --- | --- | --- |
| Rank | Match Score | Redundant Motif | P-value | log P-value | % of Targets | % of Background | Motif file |
| 1 | 0.801 | A G T C G T A C A G C T T C A G C G T A A C T G C T A G A G T C A G T C A G C T A G T C | 1e-28 | -65.107207 | 2.61% | 0.67% | motif file (matrix) |
| 2 | 0.850 | A C T G C G T A A C T G A C T G A G T C A G T C A C G T A G T C | 1e-13 | -31.260220 | 2.33% | 0.94% | motif file (matrix) |
